# Preparation and Application of Magnetic Composites Using Controllable Assembly for Use in Water Treatment: A Review

**DOI:** 10.3390/molecules28155799

**Published:** 2023-08-01

**Authors:** Yuan Zhao, Yinhua Liu, Hang Xu, Qianlong Fan, Chunyou Zhu, Junhui Liu, Mengcheng Zhu, Xuan Wang, Anqi Niu

**Affiliations:** 1 College of Chemistry and Chemical Engineering, Henan University of Science and Technology, Luoyang 471000, China; 2 College of Basic Medicine and Forensic Medicine, Henan University of Science and Technology, Luoyang 471000, China; 3 Bureau of Hydrology and Water Resources, Pearl River Water Resources Commission of Ministry of Water Resources, Guangzhou 510611, China

**Keywords:** magnetic composites, controlled assembly, water treatment, application, recycling

## Abstract

The use of magnetic composites in wastewater treatment has become widespread due to their high flocculating characteristics and ferromagnetism. This review provides an analysis and summary of the preparation and application of magnetic composites through controllable assembly for use in wastewater treatment. The applications of magnetic composites include the treatment of dye wastewater, heavy metal wastewater, microalgae suspensions, and oily wastewater. Additionally, the recycling and regeneration of magnetic composites have been investigated. In the future, further research could be focused on improving the assembly and regeneration stability of magnetic composites, such as utilizing polymers with a multibranched structure. Additionally, it would be beneficial to explore the recycling and regeneration properties of these composites.

## 1. Introduction

Due to rapid economic development and population growth, the water environment has been severely polluted by increasing domestic sewage and industrial wastewater. This has presented unprecedented challenges to water treatment. Currently, a combination of traditional physical methods (such as gravity sedimentation, physical adsorption, and membrane separation), chemical methods (including flocculation and electrochemistry), and biological methods (such as activated sludge) are widely used in water treatment [1,2,3]. Chemical flocculation is one of the techniques employed to destabilize colloids and suspended particles in water. By adding flocculants to the wastewater, the zeta-potential of the colloids and suspended particles in the water is reduced. This leads to the aggregation of these particles into larger flocs, which can then be further separated through sedimentation [4,5,6]. The process involves the continuous gathering and formation of flocs via the bridging and sweeping of the fine particles in the water [7]. As a result, the suspended particles are effectively separated and removed from the original suspensions.

Magnetic flocculation has emerged as a new method for flocculation in which magnetic flocculants are added to wastewater. The magnetic materials and colloidal particles combine to form magnetic flocs through various forces, including charge neutralization, hydrogen bonding, van der Waals forces, and others [8]. By applying an external magnetic field, the magnetic flocs can quickly aggregate and be separated from the suspensions [9]. Magnetic separation, due to its simplicity, low energy consumption, and cost-effectiveness, has found wide application in diverse water treatment processes. It has proven to be effective and reliable in various applications, such as kaolin removal, wastewater treatment in steel factories, mineral beneficiation for ore enrichment, dye wastewater decolorization, and the removal of metals like lead and copper from water [10,11,12]. Additionally, magnetic separation has found utility in biochemical processes, including cell separation, protein and DNA purification, and biocatalysis [13,14,15]. Given these versatile applications, magnetic separation solutions offer efficient and widespread usage in water treatment.

The choice of magnetic material plays a crucial role in the magnetic separation process. In recent years, various types of magnetic particles have been synthesized and have shown great potential for separation in water treatment applications. The commonly employed magnetic materials include naked Fe_3_O_4_ nanoparticles and magnetic composites that use Fe_3_O_4_ as the magnetic core [16,17,18]. However, naked Fe_3_O_4_ particles typically exhibit surface charge characteristics that are pH-dependent, with an isoelectric point around neutral or alkaline conditions. To enhance the separation efficiency, it is effective to functionalize the surface of naked Fe_3_O_4_ particles with cationic groups to prepare magnetic composites [19,20,21,22]. The cationic groups commonly used for coating purposes involve amino groups, such as polyethylenimine, poly dimethyl diallyl ammonium chloride, cationic starch, and chitosan [23,24,25,26]. The coating of a cationic polyelectrolyte was commonly achieved involving two strategies: the “attached-to” or the “immobilized-on” strategy [27,28,29]. The “attached-to” strategy involves first coating the separation objects with a polymer binder and then attaching them to the magnetic particles [22]. In the “immobilized-on” method, the naked Fe_3_O_4_ particles are first surface functionalized with a polyelectrolyte and then bound to the objects [30]. However, naked Fe_3_O_4_ particles often suffer from poor dispersibility and tend to aggregate into large clusters due to magnetostatic forces and van der Waals interactions. As a result, the “attached-to” strategy tends to achieve lower separation efficiency compared to the “immobilized-on” method [31,32,33]. Therefore, the “immobilized-on” strategy is widely applied in water treatment.

In recent years, the “immobilized-on” approach for preparing magnetic composites has gained widespread popularity. By surface functionalizing naked Fe_3_O_4_ particles, the isoelectric point of the composites generally increases, and the electrophoretic mobility also increases due to the presence of additional cationic functional groups on the surface of the magnetic particles. These particles demonstrate immense potential and efficient separation abilities in water treatment applications. This review focuses on the synthesis methods, preparation technologies, and applications of magnetic materials in the treatment of heavy metal wastewater, oily wastewater, dye wastewater, and microalgae removal or separation. This review also addresses the challenges associated with magnetic materials and discusses future developments in this field. Overall, the utilization of magnetic composites prepared using the “immobilized-on” approach holds great promise for addressing water treatment challenges. Further research and development in this area will contribute to improving the efficiency and effectiveness of magnetic materials in various water treatment applications.

## 2. Nano-Fe_3_O_4_ Particle Preparation

In the field of magnetic separation processes, naked Fe_3_O_4_ has been extensively used in water treatment due to its small size, ferromagnetism, and ease of preparation and modification. The preparation of nano-Fe_3_O_4_ primarily involves physical and chemical methods (Table 1). Physical methods such as ball milling and ultrasonic treatment are often employed. Although the operation process of this method is simple, the synthesized Fe_3_O_4_ particles frequently exhibit an uneven particle size distribution and different morphologies, making the obtained nano-Fe_3_O_4_ prone to oxidation. On the other hand, chemical methods typically involve liquid-phase reactions, which offer mild reaction conditions and a single reaction mechanism [34,35]. These methods allow for control over the nanoparticle size and result in prepared Fe_3_O_4_ particles with a uniform size distribution and good dispersion properties [16]. The commonly used chemical methods are primarily based on co-precipitation and hydrothermal synthesis.

The co-precipitation method is carried out under the protection of nitrogen or inert gas. The iron precursor (Fe^2+^ and Fe^3+^) solutions are mixed with an alkaline solution (NH_3_-H_2_O or NaOH) in a specific molar ratio at room temperature and standard atmospheric pressure. Subsequently, the Fe^2+^ and Fe^3+^ ions precipitate to form Fe_3_O_4_. After precipitation, the precipitated Fe_3_O_4_ is prepared through a series of steps, including cooling, washing, and drying [36,37,38]. The formation principle of Fe_3_O_4_ via co-precipitation involves nucleation and growth mechanisms. Although co-precipitation is advantageous in terms of its simplicity and low toxicity, rapid precipitation often leads to the agglomeration of Fe_3_O_4_ particles and an irregular particle size distribution [39]. On the other hand, hydrothermal synthesis refers to a process where an aqueous solution is enclosed in a specially sealed reactor as the reaction medium [40,41,42]. By creating high temperature and high pressure conditions externally, soluble and insoluble substances are dissolved in the environment. Subsequently, the reaction undergoes recrystallization, separation, and heat treatment to obtain nano-Fe_3_O_4_ particles [43]. Hydrothermal synthesis offers advantages such as simple operation, low-cost raw materials, high purity, environmental friendliness, and low pollution. Moreover, the resulting nano-Fe_3_O_4_ particles possess a high surface area and excellent magnetic properties at room temperature [44,45]. However, strict requirements concerning the reaction equipment and conditions arise due to the high temperatures and pressures involved in hydrothermal synthesis [46].

Regardless of the co-precipitation or hydrothermal methods, the morphology, particle size, and other physicochemical properties of the resulting nano-Fe_3_O_4_ are influenced by factors such as the reaction precursors, temperature, and reaction time [46,47]. The properties of the prepared Fe_3_O_4_ particles vary depending on the chosen preparation technology and reaction conditions. Nano-Fe_3_O_4_ particles find applications in various fields of water treatment, including suspension particle separation, heavy metal adsorption in water, and dye wastewater discoloration [48,49,50]. However, traditional Fe_3_O_4_ particles typically possess an isoelectric point between 4.0 and 6.0, with some even lower than 4.0. Consequently, in wastewater treatment scenarios with a neutral or alkaline environment, nano-Fe_3_O_4_ particles often exhibit a negative charge, resulting in low efficiency for charge neutralization and pollutant adsorption [51,52,53]. To enhance the isoelectric point of nano-Fe_3_O_4_ particles and improve their water treatment performance, naked Fe_3_O_4_ is usually functionalized with polymers before use. Cationic polymers are commonly selected to coat the surface of Fe_3_O_4_, leading to the preparation of magnetic composites that enhance the charging properties of nano-Fe_3_O_4_ [54,55]. The isoelectric point of polymer-coated nano-Fe_3_O_4_ is increased to neutral or alkaline levels, and in some cases, nanocomposite magnetic materials can reach an isoelectric point of 13.5. The elevated isoelectric point also means that more ion exchange occurs between nano-Fe_3_O_4_ and the coated polymer, which results in magnetic composites with more functional groups. This significantly broadens the applicability of nano-Fe_3_O_4_ [56,57,58].

## 3. Controlled Assembly of Magnetic Composites

### 3.1. In Situ Assembly

The current methods for the functionalized assembly of polymers with nano-Fe_3_O_4_ mainly involve two approaches: in situ assembly and ectopic assembly (Table 2). In the in situ assembly process, cationic polymers are adequately mixed with iron precursors in a one-step method within the reactor [59,60,61]. This simultaneous synthesis of nano-Fe_3_O_4_ and coating with polymers allows for the preparation of magnetic composites with different particle sizes and shapes by controlling the reaction conditions [61,62,63]. In the reactor, Fe^3+^, Fe^2+^ and the coating materials are dissolved together. Then, the magnetic composites are formed after adding the alkaline solution and washing with ultrapure water. Additionally, higher nucleation rates and improved particle size distributions can be achieved using this method. As a result, in situ assembly has been considered as one of the main directions for synthesizing magnetic composites in recent years.

**Figure 1:**
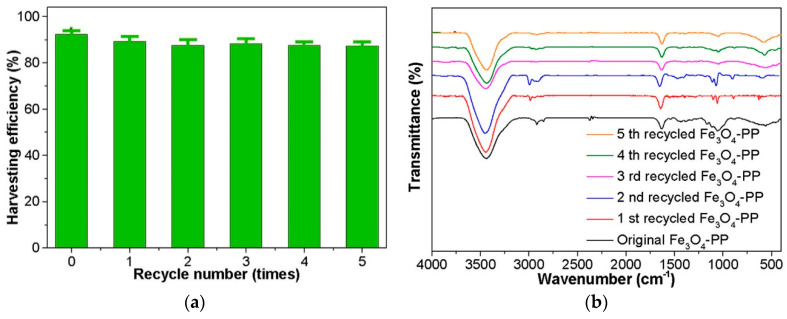
Effect of using Fe_3_O_4_/PP magnetic composites after five cycles of regeneration (**a**) and FTIR spectra (**b**) [31]. Dosage = 20.0 g/L. Initial algal cell density = 2.0 × 10^10^ cells/L. Initial pH = 9.03. n = 3.

Wang et al. incorporated plant tannins (PP) into the precursor reaction solution of Fe and synthesized the magnetic composite Fe_3_O_4_@PP hydrothermally in a high-pressure reactor [51]. The Fe_3_O_4_@PP exhibited excellent adsorption properties for microalgae harvesting (589.99 mg/g biomass) and demonstrated strong ferromagnetic properties with a hydrodynamic diameter of 287 nm. Liu used the co-precipitation method to directly add larch tannin into a mixed solution containing Fe^2+^ and Fe^3+^, resulting in the synthesis of iron-based magnetic composites in a one-step process [64]. This material showed potential in removing Cd^2+^ from wastewater. In the in situ assembly process for magnetic composite preparation, the polymer assembly and Fe_3_O_4_ synthesis occur simultaneously. This ensures that the −NH_2_ groups of the polymer can firmly coat onto the Fe_3_O_4_ structure. Furthermore, the functional groups of the polymer on the Fe_3_O_4_ surface exhibit stable retention even after repeated recycling, indicating high cyclic stability (Figure 1). After five recycles, the harvesting efficiency remains at a high level (Figure 1a). In addition, the FTIR infrared spectra show that the peak position remained unchanged during five cycles of the recycle and reused processes, which indicated that the characteristic functional groups of the materials were not damaged (Figure 1b). The particle size, shape, and stability of the magnetic composites are determined by various operating conditions, such as the concentration of polymers and iron precursors, reaction temperature, and reaction time [65,66]. However, due to the surface properties, the obtained magnetic composites often have relatively low or even negative surface charges [31,67]. Therefore, addressing the dispersion and charging properties remains a major challenge in the preparation of magnetic composites via in situ assembly.

**Table 2:** Summary of the controlled assembly of magnetic composites.

	In Situ Assembly	Ectopic Assembly		
		Ectopic Assembly with Inorganic Polymers	Ectopic Assembly with Organic Polymers	Ectopic Assembly with Biopolymers
Composition	Iron precursors + polymers	Nano-Fe_3_O_4_+ inorganic polymers	Nano-Fe_3_O_4_+ organic polymers	Nano-Fe_3_O_4_+ biopolymer
Synthesis method	One-step method	Electrostatic adherence	Coating, “grafting-to” reaction	coating, “grafting-to” reaction
Performance	Low or even negative surface charges	Strong poly-aggregation characteristics	Strong flocculation performance	Strong flocculation and adsorption performance
Samples	Fe_3_O_4_@PP [51]	PAC/Fe_3_O_4_ [68], MFPAC [69]	Fe_3_O_4_@PEI [70], Fe_3_O_4_@SiO_2_@PAMAM [71]	Fe_3_O_4_@APFS-G-CS MNPs [72]
Application	Microalgae harvesting	Microalgae harvesting, pre-concentrating waste leachate	Microalgae harvesting, graphene oxide wastewater	Oily wastewater

### 3.2. Ectopic Assembly

In contrast to the in situ assembly process, the ectopic assembly process is another common technique for preparing magnetic composites. In this approach, the nano-Fe_3_O_4_ particles, either synthesized in the laboratory or purchased directly, are assembled with cationic polymers through graft copolymerization or cross-linking modification [56,73]. The synthesis of polymer-coated nano-Fe_3_O_4_ can be conducted at room temperature without the need for high temperatures and pressures. This process is straightforward, and the experimental conditions can be easily controlled. Depending on their properties, the polymers used for ectopic assembly can be categorized as inorganic polymers, organic polymers, biopolymers, and so on [68,74,75].

#### 3.2.1. Ectopic Assembly with Inorganic Polymers

In the realm of traditional inorganic polymers, aluminum salts and iron salts are commonly used, such as polymerized ferric sulfate (PFS), polymerized ferric aluminum sulfate (PFAS), and polymerized aluminum chloride (PAC) [76,77,78]. Generally, the nano-Fe_3_O_4_ coated with inorganic polymers materials are obtained through combining with inorganic polymer solution and Fe_3_O_4_ via stirring or shaking for enough time. In contrast to conventional inorganic polymers, high-molecular-weight inorganic polymers have the ability to synergistically react with two or more polymers. This leads to the formation of larger and denser flocs, resulting in improved polymerization performance. Consequently, the functionalized assembly of inorganic polymers with nano-Fe_3_O_4_ achieves strong poly-aggregation characteristics, including adsorption, bridging, and roll sweeping effects.

For instance, Zhao et al. combined PAC with nano-Fe_3_O_4_ particles to prepare PAC/Fe_3_O_4_ magnetic flocculant, which achieved a 91% harvesting efficiency of oleaginous microalgae from the original culture suspensions. Furthermore, when PAM (polyacrylamide) was added as a coagulant aid at a concentration of 3 mg/L, the harvesting efficiency improved to 99% [68]. Liu utilized PAC as a flocculant and NaH_2_PO_4_ as a stabilizer to prepare a novel composite magnetic flocculant (MFPAC) with nano-Fe_3_O_4_ for pre-concentrating waste leachate [69]. The combination of inorganic polymers with nano-Fe_3_O_4_ effectively enhances the density and sedimentation performance of magnetic flocculants, reduces the flocculation time, and exhibits good chemical separation performance.

#### 3.2.2. Ectopic Assembly with Organic Polymers

Organic polymers, due to their effective adsorption and bridging properties, demonstrate strong flocculation performance in water treatment. When functionally assembled with Fe_3_O_4_, the resulting magnetic composites exhibit faster formation of magnetic flocs, require less hydraulic retention time, and have lower water content in the floc. These composites also maintain high magnetic separation performance even after multiple cycles of recycling and regeneration. In general, the first step in the assembly process is the synthesis of Fe_3_O_4_, which is then thoroughly mixed with organic polymers with a certain ratio. The raw materials react adequately by vibrating, ultrasound, stirring, and so on. The target magnetic composites are obtained through the external magnet and washed several times.

For example, Liu coated polyethylenimine (PEI) onto nano-Fe_3_O_4_ to obtain the magnetic composite Fe_3_O_4_@PEI [70]. This composite was used for microalgae harvesting, achieving an efficiency of over 98%. However, after 10 cycles of recycling and regeneration using the ultrasonic method, the harvesting efficiency of the Fe_3_O_4_@PEI composites reduced to 75%. Yang coated SiO_2_ on the surface of Fe_3_O_4_ nanoparticles and further modified it with polyamidoamine (PAMAM) to create a composite magnetic nano-flocculant (Fe_3_O_4_@SiO_2_@PAMAM), which exhibited excellent performance in treating graphene oxide wastewater [71].

#### 3.2.3. Ectopic Assembly with Biopolymers

Biopolymers are natural materials derived from organisms or plants, possessing characteristics such as biocompatibility, biodegradability, environmental friendliness, low cost, and non-toxicity [79,80]. These biopolymers often exhibit strong flocculation and adsorption performance in water treatment due to the presence of active groups in their structure, such as amino, hydroxyl, and carboxyl groups [81]. The functionalized assembly of biopolymers with Fe_3_O_4_ nanoparticles is commonly employed to enhance the specific surface area and adsorption capacity of magnetic composites, with remarkable stability observed in wastewater treatment applications [82].

Chitosan is a typical representative of biopolymers, which is derived from chitin and is a representative example of biopolymers [75]. Due to its strong biocompatibility, biodegradability, and abundant functional groups (–NH_2_, –OH, –COOH), chitosan is often used for functionalized assembly with nano-Fe_3_O_4_ [83,84,85]. A complexation effect easily occurs between chitosan and nano-Fe_3_O_4_ particles, facilitated by the nitrogen atoms in the amine groups of the chitosan sharing lone electron pairs with divalent or trivalent iron on the surface of the nano-Fe_3_O_4_. This enables the Fe_3_O_4_ nanoparticles to embed onto the chitosan matrix structure, forming stable magnetic composites [86].

**Figure 2:**
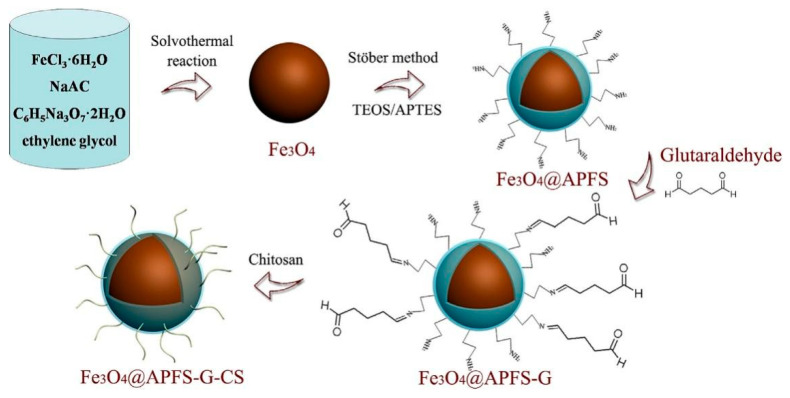
Synthetic route of Fe_3_O_4_@APFS-G-CS [72].

Lü conducted the synthesis of Fe_3_O_4_@APFS MNPs using a modified Stober method, where amino propyl-functionalized silica (APFS) and Fe_3_O_4_ nanoparticles were employed to stimulate the reaction [72]. The mixture was treated by a powerful ultrasonic wave and collected with the help of an external magnet. Subsequently, the chitosan-grafted magnetic nanoparticles (Fe_3_O_4_@APFS-G-CS MNPs) were obtained through a “grafting-to” reaction with chitosan. The synthesis procedure is depicted in Figure 2. The demulsification performance of these composites was evaluated under various conditions of oily wastewater. The results indicated that demulsification was achieved through electrostatic interactions. However, the Fe_3_O_4_@APFS-G-CS MNPs composites exhibited enhanced effectiveness for harvesting negatively charged materials (e.g., microalgae) in low pH conditions. This can be attributed to the magnetic chitosan surface being enriched with positive charges through rapid protonation [87]. Therefore, an important challenge to address is increasing the isoelectric point of chitosan-coated magnetic composites.

## 4. Application of Magnetic Composites in Water Treatment

Magnetic composites offer the combined advantages of functional polymers and magnetic properties, enabling not only efficient pollutant removal but also stable material recovery. These composites have demonstrated effective removal of pollutants from various types of contaminated water, including dye wastewater, heavy metal wastewater, and oily wastewater (Table 3). Additionally, they have proven to be efficient in separating microalgae from algal broth, catering to different processing requirements [88,89,90].

### 4.1. Decolorization of Dye Wastewater

**Figure 3:**
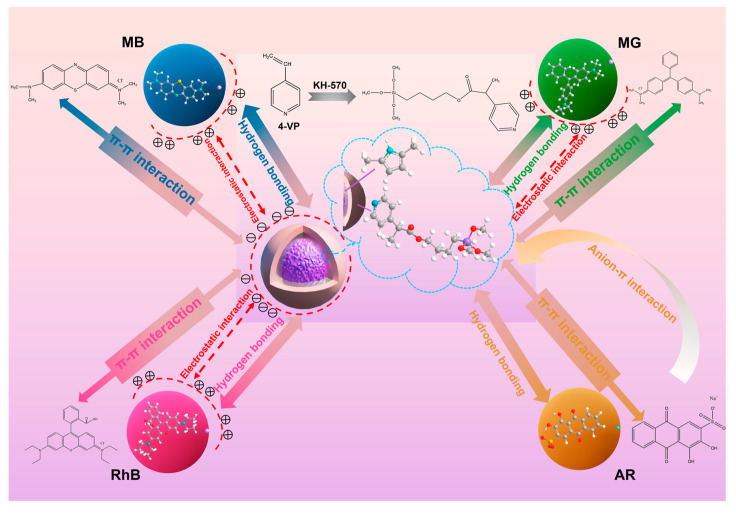
Adsorption mechanism of dyes by Fe_3_O_4_@PPy@4-VP composites [92].

The dyes in wastewater are often challenging to remove due to the presence of stable aromatic structures composed of chromophores and polar groups. Adsorption is a commonly used method for treating dye wastewater [97]. Magnetic composites, as a new type of adsorbent, possess a complex internal spatial structure that can accelerate the adsorption process of dyes. Additionally, these composites enable simultaneous material recycling and regeneration through their magnetic properties [98]. For instance, Liang employed modified Fe_3_O_4_/HA composites, which were generated from Fe_3_O_4_ nanoparticles and humic acid (HA), to remove rhodamine B (RhB) from wastewater [91]. The adsorption equilibrium for RhB by Fe_3_O_4_/HA composites was reached within 15 min, with a maximum adsorption capacity of 161.8 mg/g and a removal efficiency of ≥98.5%. The characterization results showed that the Fe_3_O_4_/HA composites aggregated after decolorization of the aqueous suspension. These composites could be rapidly recovered from the liquid using a low magnetic field after the adsorption process. Ren investigated magnetic core-shell Fe_3_O_4_@polypyrrole@4-vinylpyridine (Fe_3_O_4_@PPy@4-VP) composites for the removal of multiple dyes [92]. The results demonstrated the significant dye adsorption properties of the Fe_3_O_4_@PPy@4-VP composites. Electrostatic interactions, hydrogen bonding, and π−π interactions between the composites and dye molecules contributed to the efficient adsorption of different dyes (shown in Figure 3). Even after five cycles of adsorption–desorption, the adsorption efficiency remained unchanged. With a saturation magnetization of 33.84 emu/g, rapid separation of the Fe_3_O_4_@PPy@4-VP from the solution could be achieved.

### 4.2. Heavy Metal Removal

Magnetic composites exhibit excellent adsorption capacity for removing heavy metals, primarily due to their high specific surface area and abundant active sites. These composites, obtained through polymer assembly, possess numerous active groups that contain lone electron pairs capable of chelating with heavy metals. This allows the formation of stable chelates, enabling effective removal of heavy metals from wastewater [99]. For example, Feng prepared magnetic Fe_3_O_4_-chitosan@bentonite (Fe_3_O_4_-CS@BT) composites using natural materials and applied them in removing Cr(VI) from acid mine drainage (AMD) during remediation [93]. The results demonstrated that the Fe_3_O_4_-CS@BT had an adsorption capacity of 54.3 mg/g for Cr(VI) removal. The optimum conditions for adsorption were found to be an adsorbent dose of 0.05 g, pH 2, contact time of 120 min, initial Cr(VI) concentration of 60 mg/L, and a temperature of 25 °C. After undergoing five cycles of adsorption and desorption, the decrease in the adsorption efficiency was only 3%. Furthermore, the Fe_3_O_4_-CS@BT exhibited excellent performance in the removal of various heavy metals.

**Figure 4:**
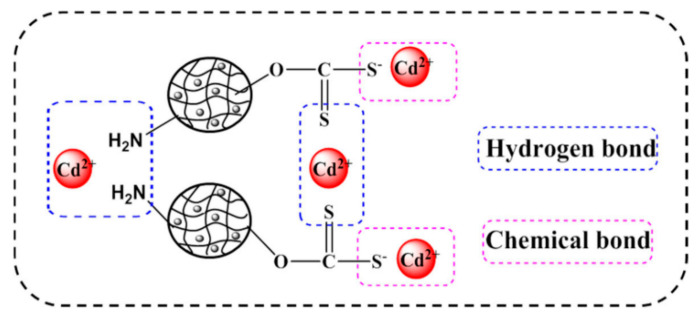
The adsorption mechanism of the XMPC for Cd(II) [94].

In Wang’s study, a magnetic xanthate-modified polyvinyl alcohol and chitosan composite (XMPC) was synthesized for the efficient removal and recovery of heavy metal ions from aqueous solutions [94]. This material was utilized to remove Pd(II), Cu(II), and Cd(II) ions. Adsorption equilibrium was achieved at 303 K after 120 min, with removal efficiencies of 67 mg/g, 100 mg/g, and 307 mg/g, respectively. The proposed mechanism for Cd(II) removal involved the gradual occupation of vacancies on the hydrogel surface by heavy metal ions as the reaction progressed (shown in Figure 4). Due to the magnetic properties of Fe_3_O_4_, the XMPC could be rapidly separated from the solution after the reaction.

### 4.3. Removal and Separation of Microalgae

The bioflocculation effect between magnetic composites and microalgae is facilitated by the electrostatic interaction, which can occur due to the porous structure, high active sites, and rich functional groups of magnetic composites. The magnetic aggregates are formed through strong hydrogen bonding between the magnetic composites and microalgae, allowing them to be easily separated from the solution using an external magnetic field [100,101]. In Ma’s research, the magnetic flocculation properties required for purifying algae-laden raw water were investigated using Fe_3_O_4_/CPAM magnetic composites [57]. It was found that the removal efficiency of chlorophyll a (Chl a) was over 97% with a Fe_3_O_4_/CPAM dosage of 1.2 mg/L, a mass ratio of Fe_3_O_4_ to CPAM of 1.5:1, and a pH range of 4.0 to 9.0. The main mechanism of flocculation involved charge neutralization at a pH of less than 9.0, while hydrogen bonding adsorption became dominant at a pH greater than 9.0.

Liu conducted a study where various materials fabricated using different methods were employed to harvest *Chlorella* sp. [102]. In the preparation of magnetic composites, Fe_3_O_4_ nanoparticles were synthesized using two methods (chemical coprecipitation and thermal decomposition) and modified with amino acids using three different approaches (ultrasonic, long-time mixing, and one step). The results revealed significant variations in oleaginous microalgae harvesting among the different materials. The amount of attached amino acid molecules on the surface of the Fe_3_O_4_ varied depending on the coating methods employed. The adsorption efficiency of oleaginous microalgae was found to be closely related to the types of amino acid groups present. Furthermore, the variation in the amount of amino acid groups on the surface of the magnetic composites influenced the harvesting performance. The structure of the amino groups also played a role in determining the harvesting efficiency. A higher number of amino groups, an increased isoelectric point, more surface active sites, and stronger electrostatic interactions and hydrogen bonding between algae cells resulted in better harvesting performance.

### 4.4. Emulsification and Separation of Oily Wastewater

In the treatment of oily wastewater, the effectiveness of the process is influenced by factors such as the van der Waals forces, oil viscosity, pore shape, and hydrophobic interactions between the oil and the adsorbent. Magnetic composites have proven to be effective in treating oily wastewater due to their high active sites, terminal functional groups, high interfacial activity, controllable viscosity, and strong ferromagnetism [103,104].

Xu’s research demonstrated the preparation of a new magnetic composite material for separating oily wastewater [95]. The process involved modifying expanded perlite (EP) with 3-aminopropyltriethoxysilane (APTES), resulting in EP@APTES. Then, Fe_3_O_4_ nanoparticles were coated onto the surface of the EP@APTES to synthesize the novel EP@APTES-Fe_3_O_4_ composite. This synthesized composite was then employed as an eco-friendly and recyclable demulsifier for emulsified oil effluent treatment. The results showed that the EP@APTES- Fe_3_O_4_ exhibited good demulsification performance and excellent salt resistance effects. These properties were attributed to the amphiphilicity of the material and the interactions between the molecules of asphaltenes and resins at the oil–water interface. Furthermore, there was no significant reduction in the separation efficiency of the EP@APTES-Fe_3_O_4_ even after four recycling cycles, indicating its potential for repeated use in the treatment of oily wastewater.

**Figure 5:**
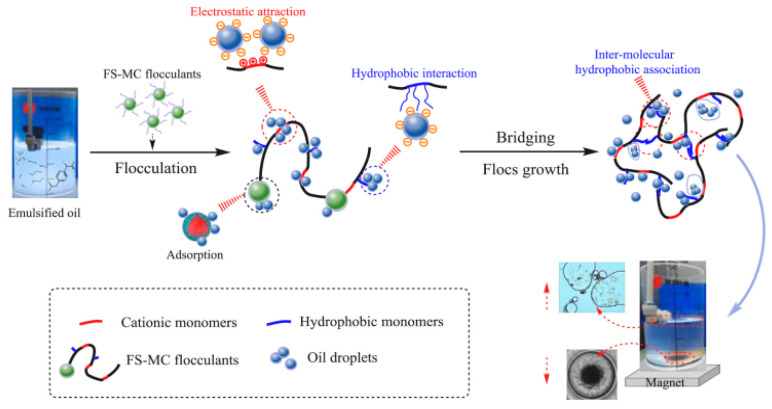
The possible flocculation mechanism of emulsified oily water treatment [96].

Ma successfully prepared a new magnetic flocculant called FS-MC by combining modified chitosan (MCS) with Fe_3_O_4_@SiO_2_ using a silane coupling agent [96]. The flocculation performance and mechanism of the FS-MC on emulsified oil wastewater were investigated in the study. The results showed that the FS-MC exhibited significant removal efficiency for organic matter with a molecular weight greater than or equal to 10 kDa. The researchers also explored the reaction mechanism of the FS-MC. It was observed that the introduction of cationic and hydrophobic groups into the FS-MC enhanced the removal efficiency of emulsified oil, as depicted in Figure 5. The potential mechanisms involved in the separation of oily wastewater included charge neutralization, compression double-layer interaction, hydrophobic interaction, interface adsorption bridging, scavenging, and other synergistic effects.

## 5. Recycling of Magnetic Composites

As the strongly ferromagnetic nature of magnetic composites allows them to be recycled and regenerated, it is necessary to determine how to collect the flocs from the obtained magnetic aggregates and evaluate the reusability of the magnetic composites after the separation process [105,106,107]. However, it is important to note that during the recycling process, there is a potential risk of releasing pollutants back into the environment. This can occur when regenerated magnetic composites are not properly controlled, leading to secondary pollution. Therefore, it is crucial to focus on selecting suitable material recycling technologies that can effectively avoid secondary pollution. The regeneration of magnetic composites employing electrostatic repulsion is conducted by adjusting the pH conditions, adding polyelectrolyte with an opposite charge, thermal regeneration or by using the ultrasound method. Due to the similarity in electrostatic repulsive forces under certain pH conditions, pH values have no significant influence on the removal efficiency of the recycled Fe_3_O_4_ particles by employing ultrasonication [4]. Therefore, the ultrasound method is adopted frequently in the recycling process of magnetic particles.

In the case of reduced graphene oxide Fe_3_O_4_ recyclable composites (RGFs), the RGFs can be recovered using a thermal regeneration method, which involves simple calcination in air at 300 °C, and then applied in methylene blue (MB) adsorption. During this process, the MB molecules undergo thermal degradation through combustion, while the Fe_3_O_4_ transforms into magnetic γ-Fe_2_O_3_ rather than non-magnetic α-Fe_2_O_3_, ensuring the materials maintain stable adsorption performance and magnetic separation properties. In recycling experiments, no significant degradation in adsorption performance was observed, indicating the potential for the effective and sustainable recycling of RGFs [108]. Thus, the advantages of the thermal regeneration method (no secondary pollution, durable adsorption performance and stable magnetic separation property) make it a favorable regeneration and recycling technique for RGFs or other similar magnetic composites.

Ghosh developed a modified magnetic nanocomposite (CDen-MNPs), which found application in two cycles of PhACs removal. The reusability of the CDen-MNPs was evaluated by conducting the adsorption–desorption process for three cycles for all pollutants, and an alcohol solution was employed as an eluent to achieve the efficient desorption of pollutants after the adsorption process. The results showed that the adsorbent could be reused at least for two cycles with consistent adsorption efficiency. Thus, the CDen-MNPs could provide a new platform for the removal of PhACs and EDCs using the magnetic separation technique, and the alcohol solution can be employed as the surfactant in the recycling process [109].

Although magnetic composites can be rapidly recycled due to their paramagnetic properties, certain types in some parts of the composites still decreased with the recycling processes, which was due to the unstable assembly of the functional groups (–NH_2_, –N^+^, –COOH, etc.) on the Fe_3_O_4_ frames [110]. Consequently, ensuring the assembly stability of recycling magnetic composites is still a significant challenge in relation to the further research and application of these materials.

## 6. Conclusions

Over the past several studies, various aspects of the process of magnetic separation in water treatment have been investigated, for instance, the synthesis of magnetic nano-Fe_3_O_4_ particles, the controlled assembled of magnetic composites, the magnetic separation process in water treatment, and the recycling and reuse of magnetic particles. With either in situ assembly or ectopic assembly, the obtained magnetic composites showed excellent potential magnetic separation efficiency with advantages such as convenience, rapid separation process, high efficiency, low energy consumption, and the reusability of the medium and magnetic particles. With the ever increasing employment of magnetic composites, their significant affects will perform more distinctly and thus render large-scale separation processes economically feasible.

## 7. Prospects

As the application of magnetic composites primarily relies on mechanisms of charge neutralization, hydrogen bonding, and covalent bonds, thus resulting in the formation of high-density magnetic flocs, so if high binding capacities are provided and high separation efficiencies are achievable, the corresponding disposal costs of the obtained flocs in the further dewatering process will be reduced significantly. Hence, it is necessary to further decrease the costs of the magnetic separation process in the future large-scale industrial application. To achieve a more effective, economic and renewable magnetic separation process, further development on some key points could be investigated in the near future:

(i) Taking assembly polymers as the starting point, to further improve the assembly stability of magnetic composites, and the regeneration and reusing properties can be promoted accordingly, thus consistent separation efficiency will be ensured.
(ii) To develop more efficient, biocompatible, degradable, highly assessable and reusable magnetic composites, which have a minimum environmental impact.
(iii) More thorough understanding of the composites–targets interactions in the magnetic separation process is essential, which could refer to the classical DLVO model and the magnetism modified M-DLVO, thus assisting in the design of magnetic composites and optimization of the magnetic separation process.
(iv) The downstream processes following magnetic separation still need to be investigated, including the re-separation of magnetic composites from the culture medium/suspensions, the extraction of desired products, etc. In addition, the design process should take the characteristic of the desired end products into account.

## Figures and Tables

**Table 1:** Summarize of nano-Fe_3_O_4_ particle preparation.

	Physical Methods	Chemical Methods
Methods	Ball milling, ultrasonic treatment	Liquid-phase reactions, hydrothermal synthesis
Advantages	Simple operation process	Mild reaction conditions, single reaction mechanism
Disadvantages	Uneven particle size distribution, different morphologies, prone to oxidation	Easy agglomeration, irregular particle size distribution

**Table 3:** Summary of magnetic composite application for water treatment.

Processing Object	Dye Wastewater	Heavy Metal	Microalgae	Oily Wastewater
Materials	Fe_3_O_4_/HA [91] Fe_3_O_4_@PPy@4-VP [92]	Fe_3_O_4_-CS@BT [93] XMPC [94]	Fe_3_O_4_/CPAM [57]	EP@APTES-Fe_3_O_4_ [95] FS-MC [96]
Targets	Rhodamine B multiple dyes	Cr(VI) Pd(II), Cu(II), Cd(II)	Algae-laden raw water *Chlorella* sp.	Emulsified oil
Mechanism	Adsorption	Adsorption, chelating	Flocculation	Flocculation
